# The Immunological Profile of SARS-CoV-2 Infection in Children Is Linked to Clinical Severity and Age

**DOI:** 10.3390/ijms24076779

**Published:** 2023-04-05

**Authors:** Claudia Vanetti, Vito Lampasona, Marta Stracuzzi, Claudio Fenizia, Mara Biasin, Irma Saulle, Fiona Limanaqi, Ahmed Abdelsalam, Cristian Loretelli, Laura Paradiso, Emma Longoni, Lucia Barcellini, Lorenzo Piemonti, Ilaria Marzinotto, Stefania Dispinseri, Antonella Amendola, Clara Fappani, Elisabetta Tanzi, Mario Salvatore Clerici, Gabriella Scarlatti, Gian Vincenzo Zuccotti, Vania Giacomet, Daria Trabattoni

**Affiliations:** 1 Department of Biomedical and Clinical Sciences, Università degli Studi di Milano, 20157 Milan, Italy; claudia.vanetti@unimi.it (C.V.);; 2 Diabetes Research Institute, IRCCS Ospedale San Raffaele, 20132 Milan, Italy; 3 Paediatric Infectious Disease Unit, Ospedale L. Sacco, 20157 Milan, Italy; 4 Department of Pathophysiology and Transplantation, Università degli Studi di Milano, 20122 Milan, Italy; 5 International Center for T1D, Paediatric Clinical Research Center Romeo ed Enrica Invernizzi, Università degli Studi di Milano, 20157 Milan, Italy; 6 Department of Paediatrics, Ospedale dei Bambini V. Buzzi, 20154 Milan, Italy; 7 Viral Evolution and Transmission Unit, IRCCS Ospedale San Raffaele, 20132 Milan, Italy; 8 Department of Health Sciences, Università degli Studi di Milano, 20133 Milan, Italy; 9 IRCCS Fondazione Don Carlo Gnocchi, 20148 Milan, Italy

**Keywords:** COVID-19, SARS-CoV-2 infection, children, immune response

## Abstract

Coronavirus disease 19 (COVID-19) is clinically less severe in children, even if the wide variety and degree of severity of symptoms reported in children pose a still-unresolved challenge for clinicians. We performed an in-depth analysis of the immunological profiles of 18 hospitalized SARS-CoV-2-infected children, whose results were compared to those obtained from 13 age- and sex-matched healthy controls (HC). The patients were categorized as paucisymptomatic/moderate (55.6%) or severe/critical (44.5%) according to established diagnostic criteria and further stratified into the categories of infants (1–12 months), children (1–12 years), and adolescents (>12 years). We assessed SARS-CoV-2-specific RBD antibodies (Ab), neutralizing antibodies (nAb), and circulating cytokines/chemokines in the plasma, and the SARS-CoV-2-specific immune response was measured in PBMCs by gene expression and secretome analyses. Our results showed peculiar circulating cytokine/chemokine profiles among patients sharing a similar clinical phenotype. A cluster of patients consisting of infants with severe symptoms presented hyperinflammatory profiles, together with extremely polarized antibody profiles. In a second cluster consisting of paucisymptomatic patients, a less pronounced increase in the level of inflammatory cytokines, together with an association between the selected cytokines and humoral responses, was observed. A third cluster, again consisting of paucisymptomatic patients, showed a circulating cytokine/chemokine profile which overlapped with that of the HC. The SARS-CoV-2-stimulated production of pro-inflammatory proteins, T lymphocyte activation, and migration-specific proteins, were significantly increased in SARS-CoV-2-infected children compared to the HC. Our findings suggest that immune response activation in the course of SARS-CoV-2 infection in children is directly correlated with clinical severity and, to a lesser extent, age.

## 1. Introduction

Children and adolescents account for 1–3% of the reported coronavirus disease 2019 (COVID-19) cases globally [1]. It is increasingly becoming accepted that COVID-19 shows a different pattern of clinical presentation in the pediatric population, with a milder disease course and overall better outcomes in comparison with adults. Nevertheless, a rare but severe evolution of SARS-CoV-2 infection in children is systemic multi-inflammatory syndrome (MIS-C), featuring an intense and dysregulated inflammatory response [2]. MIS-C remains to be fully characterized from an immune-pathogenetic point of view [3], and our understanding of the correlation between age and the immunological pathogenesis of COVID-19 is hindered by the paucity of studies comparing the immune response between adults and children. Several alternative or complementary hypotheses can be advanced to explain the differences in susceptibility and clinical spectrum severity between adults and children, as well as those between different pediatric groups. These include social contact patterns, behavior, viral loads, and age-related changes in immune and endothelial/clotting functions [4]. Moreover, the high prevalence of infections in the first years of life might play a protective role by enhancing the activation state of the innate immune system while interfering with the replication of the SARS-CoV-2 [5].

Another pivotal issue concerns the role of humoral responses in restricting the disease’s clinical course. In adults, it has been shown that patients with neutralizing antibodies (nAbs) for SARS-CoV-2 showed faster control of the virus early after infection [6], while a lack of neutralizing capacity is correlated with an increased risk of a fatal outcome.

Conversely, the role of nAbs in the clearance of an established SARS-CoV-2 infection and their persistence in pediatric patients remain to be elucidated. A few findings have shown that children with mild symptoms displayed a higher production of anti-SARS-CoV-2 nAbs compared to adults [7,8].

Dissecting the immunological profile of SARS-CoV-2-infected children with varying degrees of clinical manifestation appears to be the key to gaining a more in-depth understanding of the age-related mechanisms of susceptibility to SARS-CoV-2 and might lead to the identification of immunological biomarkers and potential molecular targets. In this study, we analyzed the clinical aspects and SARS-CoV-2-specific immunological profiles of a cohort of pediatric patients based on their clinical severity (mild/moderate and severe/critical) and age (infants, children, and adolescents). In severe cases, we also analyzed the immunological state of patients with cardiovascular involvement in comparison to those with respiratory manifestations alone.

## 2. Results

### 2.1. Clinical Characteristics of Patients and Controls

The patients were hospitalized for a mean of 11.6 days (range: 4–19 days), and only one patient was admitted to the Intensive Care Unit (ICU). Respiratory symptoms (RS: cough, rhinorrhea, wheezing, dyspnea) were present in 12 (66%) of the patients, gastrointestinal symptoms (GS: vomit, diarrhea, abdominal pain, hypoalimentation) were observed in 8 (44%), and 4 (22%) patients had both RS and GS.

We stratified the patients according to the disease severity as pauci-symptomatic or possessing moderate disease (pauci, n = 10) and severe/critical (severe, n = 8) [9]. The pauci patients had upper or lower respiratory tract infections without organ dysfunction; the severe patients required free-flow oxygen supplementation or underwent multiorgan failure. In seven children with severe disease, cardiovascular involvement, defined as a depressed left ventricular ejection fraction (EF < 55%) upon echocardiography, was present.

### 2.2. Antibody Responses of Patients and Controls

**Figure 1:**
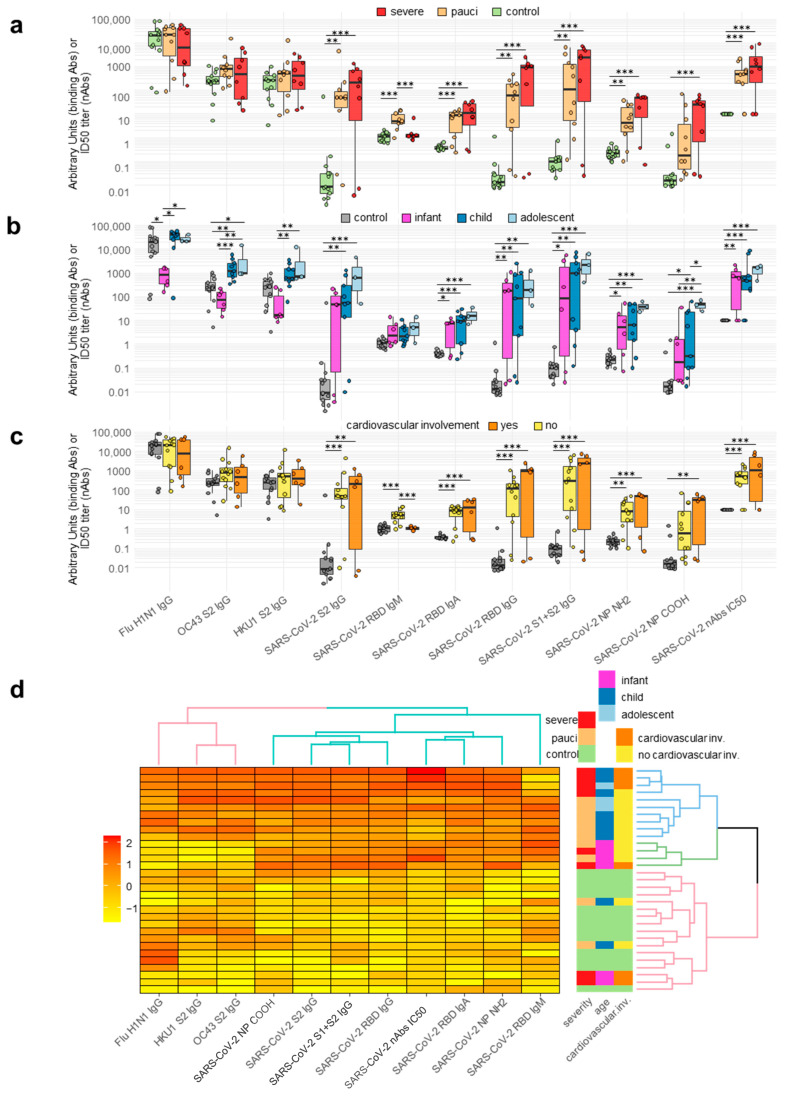
Antibody responses to pediatric COVID-19. Circles correspond to each subject’s antibody levels, expressed as arbitrary units for binding antibodies or IC50 titer for neutralizing antibodies. Value distributions are summarized as boxplots showing the median and interquartile range with whiskers extending to ±1.96-fold the median. (Panel (**a**)): Antibody levels in healthy controls (n = 13) and patients stratified by disease severity as paucisymptomatic/moderate (pauci, n = 10) and severe/critical (severe, n = 8). (Panel (**b**)): Antibody levels in patients stratified by age as infants (≤1 year, n = 6), children (1–12 years, n = 9), and adolescents (>12 years, n = 3). (Panel (**c**)): Antibody levels in patients stratified by presence (n = 6) or absence (n = 12) of cardiovascular involvement. (Panel (**d**)): Heatmap of antibody levels in study subjects, expressed using linear regression upon log transformation of the data and the clustering of patients (right panel) according to antibody levels. Significant differences between groups are indicated by asterisks (ANOVA post hoc Tukey HSD test, *p* values adjusted for multiple comparisons, *** *p* < 0.001, ** *p* < 0.01, * *p* < 0.05).

Fourteen patients (78%) had SARS-CoV-2-binding Abs and nAbs (Figure 1 and Supplementary Figure S1). The binding Abs were measured as IgG in 14 cases (78%), IgA in 13 cases (72%), and IgM in 10 (56%) cases. The four children without detectable SARS-CoV-2 spike antibodies included both pauci and severe patients and were tested within 8 days after hospitalization. None of the controls had detectable antibodies for SARS-CoV-2 spike or NP. As expected, the mean levels of binding and neutralizing Abs for SARS-CoV-2 were significantly increased compared to the controls (*p* adjusted range <0.05–<0.001). Few differences in Ab levels between the patients, having been stratified by disease severity and age, reached statistical significance. The RBD IgGs were higher in severe vs. pauci children (*p* adjusted <0.05), RBD IgMs were increased in pauci adolescents vs. severe infants (*p* adjusted <0.05), and nAbs were increased in severe children vs. infants (*p* adjusted <0.05) (Figure 1a,b and Supplementary Figure S1). No difference in antibody responses to SARS-CoV-2 were detected between patients with or without a cardiovascular involvement (Figure 1c and Supplementary Figure S1).

IgGs for the spike S2 protein of the seasonal beta-coronaviruses OC43 and HKU1 were present in all but three of the children (one control and two pauci infant patients), and IgGs for the HA Flu H1N1 were present in all of the study subjects. Among the patients, seasonal beta-coronaviruses IgGs were higher in children vs. infants with either severe or pauci disease. IgGs for the flu H1N1 HA were lower in infants compared to child (1–12 years) and adolescent patients.

Clustering of the antibody responses showed the presence of two major patient subgroups. The first was broad and heterogenous in terms of age and disease severity, showing strong responses against multiple SARS-CoV-2 and seasonal beta-coronavirus antigens; the second included controls and four patients without SARS-CoV-2 antibody responses (Figure 1d).

### 2.3. Cytokine and Chemokine Plasma Levels in Pediatric Patients

The plasma concentrations of cytokines and chemokines were measured in 14 patients and 11 controls. Severe patients (n = 6) showed a significant increase in a subset of plasma cytokine/chemokine levels (IL-2, IL-4, IL-6, IL-8, MIP-1β, and TNF-α; ANOVA, *p* adjusted <0.05 to <0.001) in comparison with the pauci COVID-19 patients (n = 8) and/or controls (Supplementary Figure S2). Several plasma cytokines/chemokines showed a trend towards increased plasma levels in the infant patients, which reached statistical significance for MIP-1β, TNF-α, and IL-2 (ANOVA, *p* adjusted <0.05) (Supplementary Figure S2). The stratification of COVID-19 patients according to the presence or absence of cardiovascular involvement (CI) showed a trend towards increased plasma levels of several plasmatic cytokines/chemokines, which reached statistical significance for IL-1 β, IL-2, IL-4, IL-6, IL-7, IL-13, and IL-17 (ANOVA, *p* adjusted <0.01 to <0.001) (Supplementary Figure S2). The stratification of patients based on both age and disease severity showed that the highest plasma levels of cytokines and chemokines were almost invariably found in infant patients with severe disease (Supplementary Figure S3).

**Figure 2:**
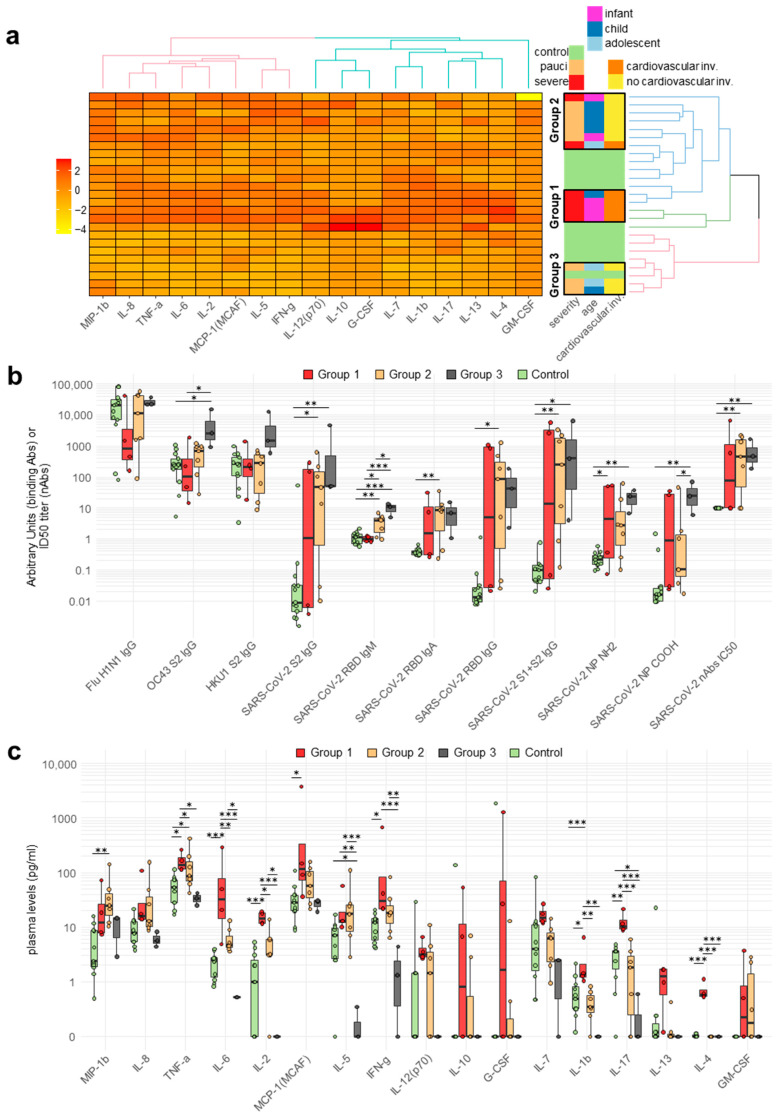
Plasma cytokine/chemokine levels in pediatric COVID-19 patients. (Panel (**a**)): Heatmap of cytokine/chemokine levels in study subjects expressed using linear regression upon log transformation of the data and clustering (right panel) of the patients according to cytokine/chemokine plasma levels. (Panel (**b**)): Antibody levels in patient groups derived from the clustering in (panel (**a**)). Panel (**c**): Cytokine/chemokine plasma levels in patient groups derived from the clustering in (panel (**a**)). In (panel (**b**,**c**)), circles correspond to each subject’s antibody levels or cytokine/chemokine plasma concentration. Value distributions are summarized as boxplots showing the median and interquartile range with whiskers extending to ±1.96-fold the median. Significant differences between groups are indicated by asterisks (ANOVA post hoc Tukey HSD test, *p* values adjusted for multiple comparisons, *** *p* < 0.001, ** *p* < 0.01, * *p* < 0.05.

Clustering of the patients according to their cytokine/chemokine plasma levels showed the presence of three major subgroups (Figure 2a). Group 1 included three infants and one child, all with severe disease and CI, with two patients showing strong SARS-CoV-2-binding and -neutralizing Abs (2/4, 50%) (Figure 2b), both sampled in the early acute phase of the disease. Group 1 was characterized by a hyper-inflammatory profile with the elevation of several cytokines/chemokines, including IL-1 β, IL-2, IL-4, IL-6, IL-7, IL-13, IL-17, IFN-γ, and TNF-α (Figure 2c). Group 2 included patients of heterogeneous age who were mostly paucisymptomatic and, save for a single exception, without CI, showing a higher prevalence of nAbs (5/7, 71%) and a less generalized increase in the levels of plasmatic cytokines/chemokines (Figure 2a–c). Group 3 included one paucisymptomatic child and two adolescent patients who were positive for nAbs but had a plasma cytokine/chemokine profile that essentially overlapped with that of the controls (Figure 2a–c).

### 2.4. Correlation between Circulating Antibodies and Cytokine/Chemokine Responses of Patients

We analyzed the correlation between antibody and cytokine/chemokine responses in the patients, grouped according to the previous cytokine/chemokine clustering. In patients belonging to group 1, i.e., infant or child patients with severe disease and CI, there was a positive correlation between binding and neutralizing SARS-CoV-2 Abs, as well as a correlation between SARS-CoV-2 Abs and seasonal beta-coronavirus Abs (Supplementary Figure S4). In particular, the correlation was high and significant (*p* not adjusted) between nAbs, SARS-CoV-2 RBD Abs of the IgA class, and IgG against the HCoV-OC43 spike protein. In group 1, we consistently observed a strong positive correlation within two subsets of cytokines/chemokines: the first subset included IL-1β, IL-2, IL-5, IL-6, IL-8, GM-CSF, IFN-γ, and MCP-1, while the second subset included IL-4, IL-17, and IL-13. Overall, in group 1, there was a negative correlation between antibody and cytokine/chemokine responses, particularly between IL-2 and SARS-CoV-2-binding Abs, with the caveat that the two Ab-negative infants were sampled at the time of acute disease presentation, i.e., likely too early for the development of SARS-CoV-2-specific antibodies.

Group 2 included two infants, four children and one adolescent patient, who were mostly pauci-symptomatic. In this group, we observed a strong correlation of SARS-CoV-2 IgA and IgG RBD Abs with nAbs. The correlation between the seasonal beta-coronaviruses and SARS-CoV-2 Abs was instead weak or negative and did not reach statistical significance (Supplementary Figure S5). In group 2, correlations of several chemokine/cytokine responses were consistently present, but they reached statistical significance only in the case of a few subsets, such as the correlation between IL-1β, IL-8, MIP1-β, and TNF-α, that between IL-13 and G-CSF, and that between IL-5 and IFN-γ. SARS-CoV-2 IgG Abs were negatively correlated with IL-12, while seasonal beta-coronavirus IgGs were negatively correlated with IL-8 and TNF-α.

Group 3 included one child and two adolescent pauci patients. Group 3 was characterized by extremely low plasma levels of most circulating cytokines/chemokines. The correlations between antibodies and cytokines/chemokines were weaker and rarely significant (Supplementary Figure S6). In particular, we observed positive correlations between IgG and IgA RBD Abs, between seasonal beta-coronavirus Abs, and between IL-7 and MIP-1β. Negative correlations were found between SARS-CoV-2 IgG Abs and IL-8 and between IL-5 and MCP-1.

### 2.5. SARS-CoV-2-Specific Cytokine/Chemokine Secretion

**Figure 3:**
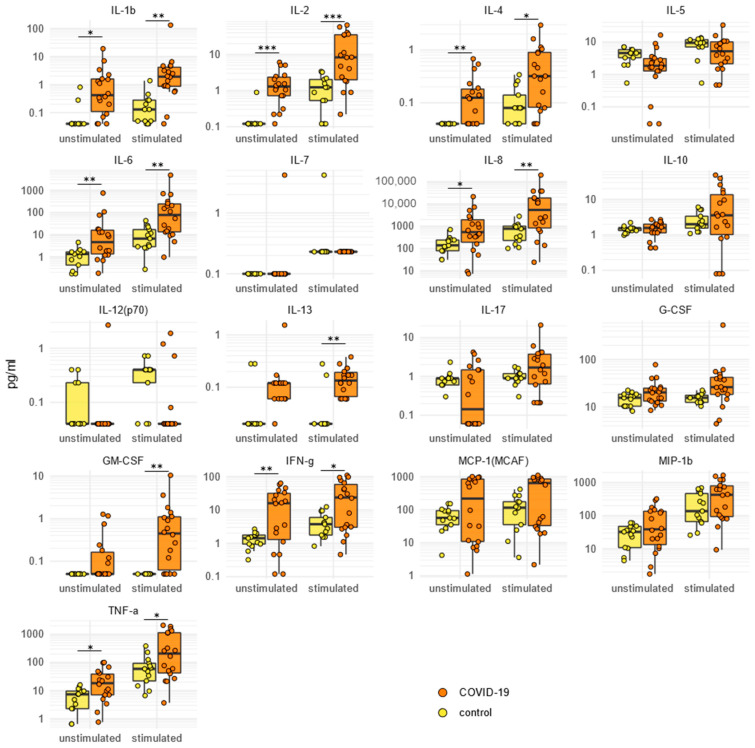
Cytokine and chemokine secretion by PBMCs upon SARS-CoV-2-antigen-specific stimulation. For each indicated cytokine or chemokine, results are shown for the control and patient secretions using unstimulated or SARS-CoV-2-stimulated PBMCs. Circles correspond to the levels of cytokines/chemokines secreted by the PBMCs of each subject. The value distributions are summarized as boxplots showing the median and interquartile range with whiskers extending to ±1.96-fold the median. Significant differences between patient and control or unstimulated vs. stimulated secretions are indicated by asterisks (post hoc Tukey HSD test after repeated-measure ANOVA, *p* adjusted, *** *p* < 0.001, ** *p* < 0.01, * *p* < 0.05).

We compared the patients (n = 18) and controls (n = 13) in terms of cytokine/chemokine secretion by PBMCs stimulated in the presence/absence of SARS-CoV-2 antigens. In the absence of virus-specific stimulation, the secreted levels of IL-1β, IL-2, IL-4, IL-6, IL-8, IFN-γ, and TNF-α were statistically significant higher in the SARS-CoV-2-infected patients compared to the HCs (repeated-measure ANOVA, *p* adjusted range <0.05 to <0.001) (Figure 3). Stimulation with SARS-CoV-2 antigens led to a significantly higher secretion of IL-1β, IL-2, IL-4, IL-6, IL-8, IL-13, GM-CSF, IFN-γ, and TNF-α in the patients compared to the controls (Figure 3).

Clustering of the cytokine/chemokine secretion levels before and after stimulation showed the presence of two major, broadly overlapping groups of patients, characterized by their heterogenous clinical and demographic features and differing mainly in their levels of secreted cytokines/chemokines (Supplementary Figure S7).

### 2.6. SARS-CoV-2-Specific Immune Gene Expression

**Figure 4:**
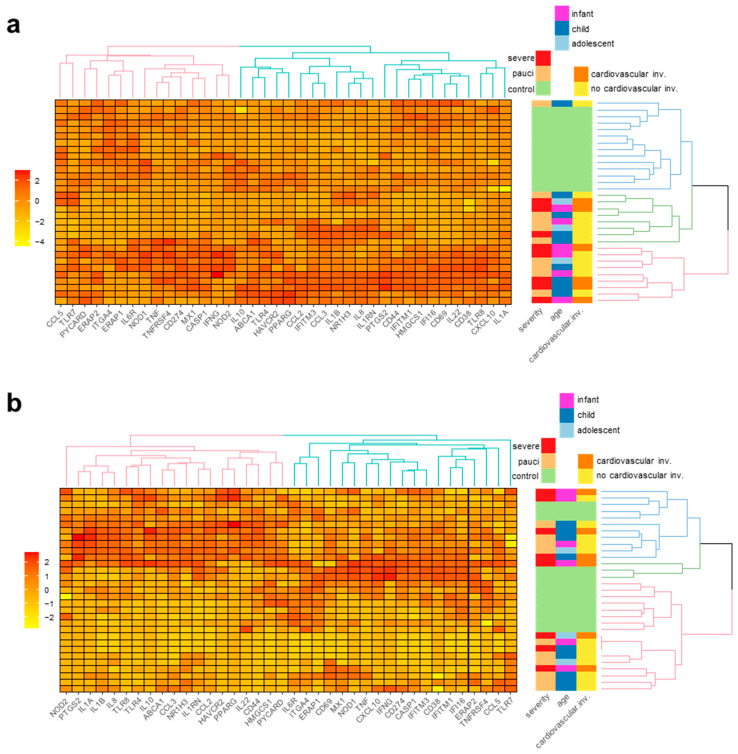
SARS-CoV-2-specific gene expression in PBMCs upon SARS-CoV-2-antigen-specific stimulation. Heatmap of gene expression levels in study subjects expressed using linear regression upon log transformation of the data and clustering (right panel) of the patients according to gene expression levels in the control and patient unstimulated (Panel (**a**)) or SARS-CoV-2-stimulated PBMCs (Panel (**b**)).

We analyzed the mRNA expression of 70 genes involved in the antiviral immune response in unstimulated and SARS-CoV-2-stimulated PBMCs from all the enrolled subjects. The results showed that 38 genes were modulated upon stimulation. The unstimulated gene expression clustered the subjects clearly, discriminating the patients from the controls (Figure 4a).

The patients’ stratification according to disease severity showed a trend towards increased mRNA levels for several genes that reached statistical significance in the cases of IL-1A, IL1B, IL1RN, and PTGS2 in severe patients compared with the controls (ANOVA Tukey post hoc test, *p* adjusted <0.05 to <0.001) (Supplementary Figure S8). ITAG4 was the only gene whose mRNA was significantly decreased in patients compared to the controls (pauci vs. controls, ANOVA Tukey post hoc test, *p* adjusted <0.01). After stratification into age groups, the genes that showed statistically significant differences in mRNA levels between the selected patient age groups and controls were IL1B, IL6R, IL1A, IFI16, CCL3, and IL8 (ANOVA Tukey post hoc test, *p* adjusted <0.05 to <0.01) (Supplementary Figure S8). ITAG4 expression was decreased in the infant patients (ANOVA Tukey post hoc test, *p* adjusted <0.05). Patients with and without cardiovascular involvement were not segregated into distinct clusters (Figure 4a). However, the stratification of the patients based on cardiovascular involvement showed a few statistically significant differences compared to the controls in regard to the mRNA levels of the following genes: IL1B and IL1A (COVID-19 with cardiovascular involvement vs. controls, ANOVA Tukey post hoc test, *p* adjusted <0.05), as well as IL1B, NR1H3, IL1RN, and ERAP1 (COVID-19 without cardiovascular involvement vs. controls, ANOVA Tukey post hoc test, *p* adjusted <0.05) (Supplementary Figure S8). ITAG4 expression was decreased in the patients without cardiovascular involvement vs. the controls (ANOVA Tukey post hoc test, *p* adjusted <0.01).

The modulation of SARS-CoV-2 antigens modulated gene expression in both the patients and controls. Clustering of the gene expression levels led to three patient groups that were heterogeneous in terms of disease severity, age, and the presence of cardiovascular involvement (Figure 4b). The stratification according to disease severity showed few statistically significant differences in SARS-CoV-2-stimulated gene expression between the severe COVID-19 (ERAP1 and IFI16, ANOVA Tukey post hoc test, *p* adjusted <0.05) and pauci COVID-19 patients (ERAP1, CD44, ANOVA Tukey post hoc test, *p* adjusted <0.05) compared to thew controls (Supplementary Figure S9). ITAG4 gene expression was also significantly decreased in the stimulated PBMCs from severe patients compared to the controls (ANOVA Tukey post hoc test, *p* adjusted <0.05). The stratification according to age showed a statistically significant difference in the stimulated mRNA levels only for ERAP1 (adolescent COVID-19 patients vs. controls, ANOVA Tukey post hoc test, *p* adjusted <0.05) (Supplementary Figure S9). The patients’ stratification according to cardiovascular involvement showed a significant difference in the stimulated mRNA levels for ERAP1 (COVID-19 patients with cardiovascular involvement vs. controls, ANOVA Tukey post hoc test, *p* adjusted <0.05) and ERAP1, as well as CD44 (COVID-19 patients without cardiovascular involvement vs. controls, ANOVA Tukey post hoc test, *p* adjusted <0.05) (Supplementary Figure S9). ITGA4 gene expression was lower in cases without cardiovascular involvement compared to controls (ANOVA Tukey post hoc test, *p* adjusted <0.05).

## 3. Discussion

It is now well recognized that, in contrast to adults, children infected with SARS-CoV-2 are usually spared from severe respiratory disease. However, in the fraction of children who develop symptomatic COVID-19, the immunopathogenesis is still only partially understood. Our work provides a deep immunological characterization of the antibody responses and chemokine/cytokine profiles of a cohort of hospitalized COVID-19 pediatric patients, including infants and adolescents with a spectrum of clinical severities. Of note, in our study, we considered severe cases eventually presenting cardiovascular involvement without multiorgan failure. Thus, none of the included patients fitted with the WHO or CDC criteria for MIS-C diagnosis [10,11].

In pediatric COVID-19, the roles of patients’ SARS-CoV-2 antibodies in clearing the infection and determining specific clinical outcomes are not fully understood [12]. While in adults, there is evidence that the early SARS-CoV-2 nAb response is inversely correlated with COVID-19 disease severity [6], in children, an efficient viral clearance may not depend on a strong antibody response [13,14]. A limited number of previously published studies have reported the presence of both binding and neutralizing antibody responses in children and adolescents with mild or asymptomatic SARS-CoV-2 infection [7,15,16]. Here, our results show that in some cases, even among patients of a similar age and disease severity, SARS-CoV-2 antibody responses can exhibit different phenotypes. This could be attributed to known age-associated differences in B cell development [17], data that must be confirmed and supported by further, future investigations based on a larger sample size. For instance, among the infants with severe disease, some cases presented without detectable binding Abs or nAbs, while others showed very high levels of both. Intriguingly, in almost all our COVID-19 patients, we observed high levels of IgG for the S1/S2 proteins of two seasonal hCoVs (HKU1 and OC43). This might be connected with either the maternal transmission of antibodies or prior exposure to seasonal beta-coronaviruses. In contrast with Aydillo T et al. [18] and Shrock E. et al. [19] and in agreement with Sasson J. M. et al. [20], we found a positive correlation between SARS-CoV-2 and seasonal human beta-coronavirus antibodies, suggesting likely cross-activation by SARS-CoV-2 due to a pre-existing humoral immune response against seasonal hCoVs, as previously described [21].

It has been suggested that pediatric COVID-19 patients might be characterized by a different profile of circulating inflammatory cytokines and chemokines compared to adults. Critical and fatal COVID-19 outcomes in adults have been associated with elevated levels of mostly IL-6, IL-1β, and TNF-α, along with a reduced and delayed production of type I IFNs [22,23,24,25]. Upon the stratification of our cohort on the basis of disease severity, elevated concentrations of pro-inflammatory cytokines/chemokines in the plasma were found in severe cases (i.e., IL-2, IL-4, IL-6, IL-8, MIP1-β, and TNF-α). This was more evident specifically in infants presenting with severe disease, showing a picture suggestive of a hyper-inflammatory profile that is presumably correlated with a cytokine storm. Our findings strengthen the data from several reports showing that infants of or below 3 months of age are mostly likely to experience severe symptoms of COVID-19 [26], with young infants (patients less than one year of age) and young adults (patients >15 years of age) most likely to require hospitalization [27]. Among infants displaying severe COVID-19 and presenting with pneumonia or gastrointestinal symptoms, high levels of IL-6 were found [28].

We further investigated the correlation of cytokines with the COVID-19 phenotype by clustering the subjects in our cohort based on age classes, disease severity, and plasma cytokines. Subgroups of patients sharing similar clinical phenotypes showed peculiar circulating cytokine/chemokine profiles. This was particularly evident in a cluster of patients consisting of infants with cardiovascular involvement, who presented hyperinflammatory profiles with significantly elevated plasma IL-2, IL-6, MIP1-β, and TNF-α levels. While there are contrasting findings on the role of IL-6 in pediatric COVID-19 [28,29,30], in adults, IL-6 has been reported to be a relatively good predictor of disease severity [31]. Our severe infant patients presented with elevated circulating IL-6 levels similar to those of adults with COVID-19, suggesting that high IL-6 levels might also constitute a signature of severe COVID-19 in children. In this cluster of patients, the SARS-CoV-2 antibodies showed extremely polarized profiles, with some patients being completely negative for binding and neutralizing antibodies, while others showed very high levels of both. Technical issues are an improbable cause of these contrasting observations, since the binding antibodies were determined using validated assays based on alternative SARS-CoV-2 antigens (whole spike, spike RBD, and nucleocapsid proteins). Although the measurements of different Ig classes did not show the presence of early IgM vs. later IgG responses in these polarized cases, the variable timing of sampling of these children during hospitalization, i.e., in an acute vs. sub-acute disease phase, might have influenced the results. Of note, all infants in whom Abs were not detected were sampled in the acute phase of the disease, within 7 days following hospitalization, i.e., likely too early for the development of SARS-CoV-2-specific antibodies. Interestingly, in this cluster, we consistently observed an inverse correlation between the levels of inflammatory cytokines/chemokines and those of antibodies for multiple SARS-CoV-2 antigens. Therefore, it might be speculated either that a rapid progression of disease can occur before antibody development or that early, high cytokine levels might actually impair the rapid establishment of a strong humoral response, particularly in the context of the immature immune system of infants. Circulating cytokines/chemokines and antibodies highlighted the presence of another distinctive patient cluster characterized by paucisymptomatic COVID-19 without cardiovascular involvement, in whom the increase in the levels of inflammatory cytokines was less pronounced and more heterogeneous, and there was an association between certain cytokine and humoral responses, particularly between SARS-CoV-2 IgG Abs and IL-12 and between seasonal beta-coronavirus IgG, IL-8, and TNF-α. In this group, we consistently observed strong correlations of IL-5 with IFN-γ and IL-1β with TNF-α, supporting the hypothesis of synergistic Th1 and Th2 responses and cooperative activity between type 1 and type 2 macrophages in controlling COVID-19. In addition, the observed inverse correlation between IL-6 and IL-17 in these patients might be understood as an attempt to dampen and avoid excessive inflammatory responses. Finally, we detected a third cluster of paucisymptomatic patients with circulating cytokine/chemokine profiles that substantially overlapped with those of the healthy controls, a finding that is in agreement with previous reports on children with mild COVID-19 and likely reflects a lower level of inflammation [32]. The most prominent feature distinguishing these two clusters of mild/paucisymptomatic patients was the presence or absence of a strong correlation between the cytokine and SARS-CoV-2 Ab levels. It could be speculated that a coordinated increase in both cytokine and Ab responses might mitigate disease severity.

Elevated concentrations of cytokines/chemokines were secreted by the SARS-CoV-2 pediatric patient PBMCs vs. the control cells, regardless of disease severity or age, and this was true both with and without SARS-CoV-2-specific stimulation. Similarly, the profiling of the immune gene transcripts in the PBMCs revealed a generalized upregulation of multiple inflammatory factors in the patients that was particularly evident in severe cases. Interestingly, we detected a decreased level of ITGA4 (CD49d) mRNA in the severe patients, including those with cardiovascular involvement. This finding is consistent with the reported decrease in the levels of CD49d in adults with severe COVID-19 [33], suggesting that CD49d might also be a biomarker of worse disease progression in pediatric patients.

Our study has some limitations, particularly in regard to the limited number of enrolled patients and the heterogeneity of the timing of sampling with respect to the time of symptoms onset. Nevertheless, we believe that it provides, for the first time, intriguing results with regard to the correlation between cytokine/chemokine and Ab responses in pediatric patients with different clinical manifestations of COVID-19.

Our data support the existence of specific immunological profiles correlated with disease severity and age in pediatric patients that can easily be analyzed using peripheral blood samples. Considering that a non-negligible fraction of the pediatric population remains susceptible to the development of serious health consequences upon SARS-CoV-2-infection, our results deserve replication in larger cohorts and contribute to our understanding of the immunological factors associated with severe COVID-19 in children, a goal that remains fundamental in order to inform prevention and mitigation strategies. However, we can speculate that a given immune profile may help to detect a particular underlying clinical condition and may be useful for better managing the evolution of COVD-19 in pediatric patients.

## 4. Materials and Methods

### 4.1. Patient Population

We conducted a multicenter prospective analysis of clinical records and biological samples of SARS-CoV-2-infected children hospitalized from 21 February to 1 May 2020 at the Luigi Sacco Hospital and Vittore Buzzi Hospital, Milan, Italy. The study was approved by the ethical committee of the coordinating center in Milan (protocol number 2020/ST/061). The humoral immunity study was approved by the IRCCS Ospedale San Raffaele Ethics Review Board (protocol number 34/INT/2020). All research was performed in accordance with the relevant regulations, and informed consent was obtained from all the participants’ parents or their legal guardians. The study was performed in accordance with the Declaration of Helsinki. SARS-CoV-2 infection was based on either a positive result of a SARS-CoV-2 real-time reverse-transcriptase PCR (RT-PCR) assay based on a nasal/pharyngeal swab specimen or the presence of serum anti-SARS-CoV-2-specific antibody IgG using a semi-quantitative anti-SARS-CoV-2 ELISA (Euroimmun, Lübeck, Germany). Data regarding SARS-CoV-2 exposure history, clinical symptoms or signs, and laboratory findings upon admission were collected using a standardized form. We enrolled 18 children (12 females and 6 males) with a mean age of 6.1 years (range 0.1–14.7 years) (Table 1). The patients were stratified according to age into 3 groups: 7 infants (1–12 months), 8 children (1–12 years), and 3 adolescents (>12 years). As a control group, we included 13 sex- and age-matched children (HC). None of the patients or the controls had a pre-existing medical condition.

The specimens for the immunological studies were stratified in two groups according to time following hospitalization (cut-off 7 days).

### 4.2. SARS-CoV-2, Seasonal HCoVs, and H1N1 Flu Virus Antibody Assays

Serum samples of 18 children were tested for antibody responses to SARS-CoV-2, seasonal beta-coronaviruses, and the 2009 pandemic H1N1 influenza virus. The detection of IgM, IgG, and IgA antibodies binding to the spike S1/S2 and spike RBD antigens of SARS-CoV-2, IgG binding to the spike S2 antigens of HCoV-HKU1 and HCoV-OC43, and IgG binding to the hemagglutinin antigen (HA) of the 2009 A/H1N1 influenza virus, was performed by LIPS, as previously described [34]. Briefly, each recombinant nanoluciferase-tagged antigen, expressed in transfected eukaryotic cells, was incubated in liquid phase with test sera for 2 h, followed by capture of the immune complexes with rProtein A-sepharose, washing to remove the unbound antigens, and the measurement of the recovered luciferase activity using an XS960 Centro luminometer (Berthold Technologies, EG & G Co, Wildbad, Germany). Using Ab-positive sera as standards, the raw data were then converted into arbitrary units (AU).

nAbs were measured using a neutralization assay based on a lentiviral vector (LV) expressing the SARS-CoV-2 spike protein and a luciferase reporter, as previously described 6. Briefly, serum serial dilutions were incubated with the virus for 30 min at 37 °C and then added to VeroE6 cells. After 48 h, the luciferase expression was determined using a luciferase assay system (Bright-Glo, Promega Co., Madison, WI, USA) and measured using a Mitras luminometer (Berthold Technologies, EG & G Co, Wildbad, Germany). The inhibitory serum dilution (ID) 50 was calculated using a linear interpolation method [35].

### 4.3. Isolation and Stimulation of Peripheral Blood Mononuclear Cells (PBMCs) with SARS-CoV-2-Specific Antigens

PBMCs were isolated from whole blood by density gradient centrifugation using Ficoll (Cedarlane Laboratories Limited, Hornby, ON, Canada), as previously described [36], and the viable cells were counted with the cell counter ADAM-MC (Digital Bio, NanoEnTek Inc., Seoul, Korea). The PBMCs were collected at a concentration of 1 × 106/mL in RPMI 1640 medium (Euroclone, Milan, Italy), 10% Fetal Bovine Serum (FBS), 1% of L-glutamine (LG), and 2% penicillin/streptomycin. Next, the PBMCs were challenged with 500 ng/mL nucleocapsid (N)- and spike (S)-specific SARS-CoV-2 antigens (Novatein Biosciences, Woburn, MA, USA). For the gene expression and cytokine analyses, the cells were harvested after 10 h of specific stimulation, and the cytokines/chemokines were quantified in cell culture supernatants.

### 4.4. Quantigene Plex Gene Expression Assay

Gene expression analysis was performed using a QuantiGene Plex assay (Thermo Scientific, Waltham, MA, USA), following 10 h of stimulation of 8 × 105 PBMCs with SARS-CoV-2 antigens. The QuantiGene Plex Assay allows for the simultaneous quantification of the expression of 70 selected genes involved in the antiviral immune response and 4 housekeeping genes (Table 2).

### 4.5. Multiplex Cytokine Analyses

A 17-cytokine multiplex assay was performed on plasma and cell culture supernatants from patients 10 h after PBMC stimulation with SARS-CoV-2-specific antigens, as described above, using a multiplexed Luminex magnetic bead immunoassay (Bio-Rad, Hercules, CA, USA) according to the manufacturer’s instructions.

### 4.6. Statistical Analysis

To describe the continuous variables, median values with interquartile ranges (IQR) were applied, whereas frequencies in percentages and counts were used for the categorical variables. To compare the variables, the chi-square or Fisher’s exact test was used for the categorical variables, and the Wilcoxon rank sum test was used for the continuous variables. An ANOVA with Tukey’s HSD post hoc test was applied for comparisons of the antibody, cytokine, and chemokine levels between groups. The correlations between the antibody, cytokine, and chemokine levels were assessed using linear regression upon log transformation of the data. For the Tukey’s HSD post hoc analyses, two-tailed *p* values adjusted for multiple testing are reported, with a *p* value < 0.05 considered statistically significant. Statistical analyses were performed using the R software version 3.4.0 (R Core Team (2017). R: A language and environment for statistical computing. R Foundation for Statistical Computing, Vienna, Austria. https://www.R-project.org/ (accessed on 22 July 2022)).

## Figures and Tables

**Table 1:** Clinical characterization of pediatric patients and controls enrolled in the study.

	Patients		Controls	
	#	%	#	%
Median age (range) in years	6.1 (0.1–14.7)	-	9.4 (1.0–16.6)	-
Female	12	33	5	38.5
Comorbidities	0	0	0	0
White cell count (average ± SD)	8071 ± 3506		-	-
Neutrophils (average ± SD)	2962 ± 2234	35 ± 15.4	-	
Lymphocytes (average ± SD)	4215 ± 1976	53.2 ± 14.8	-	-
Positive RT-PCR based on respiratory specimen	13/19	68.4	-	-
IgG Anti-SARS-CoV-2	11/13	84.6	-	-
Paucisymptomatic/moderate (PM)	10	55%	-	-
Severe/critical (SC)	8	45%	-	-

**Table 2:** Genes involved in the antiviral immune response and housekeeping genes quantified by the QuantiGene Plex Assay.

IFNA2	CASP1	CD209	TAP1
IL2	ACTB	IL1B	MPO
IL28A	TLR4	IL10	NLRP3
IL17A	IL18	IL1RN	IL6R
CCL2	IL7	ABCA1	HAVCR2
TNFRSF4	PTGS2	ELOVL6	CXCL10
IFNB1	MX1	CD38	IFNG
PPARG	IFITM3	IL12B	NOD2
CRP	CD44	NOS2	CCL3
PPIB	CLEC4M	CH25H	CD69
COVID19	IFITM1	IFI16	AGTR1
CCL5	CD274	ACAT1	ERAP1
GAPDH	IL1A	HPRT1	TBP
PDCD1	IL12A	NOD1	ERAP2
HMGCS1	ITGA4	CSF3	
TLR8	NR1H3	AGTR2	
IL22	IL8	TLR3	
CSF2	ITGB7	IL6	
PYCARD	TLR7	ACE	
TNF	ACE2	TMPRSS2	

## Data Availability

The data that support the findings of this study are available upon request from the corresponding author, M.S. The data are not publicly available because they contain information that could compromise the privacy of the research participants.

## Supplementary Materials

The following supporting information can be downloaded at: https://www.mdpi.com/article/10.3390/ijms24076779/s1.
