# Supplementary material for: The Immunological Profile of SARS-CoV-2 Infection in Children Is Linked to Clinical Severity and Age
Source: Int J Mol Sci. 2023 Apr 5;24(7):6779. doi: 10.3390/ijms24076779 (PMC10095251; doi:10.3390/ijms24076779)
Supplement: Supplementary file 1 [file ijms-24-06779-s001.zip › ijms-2264867-supplementary.pdf]

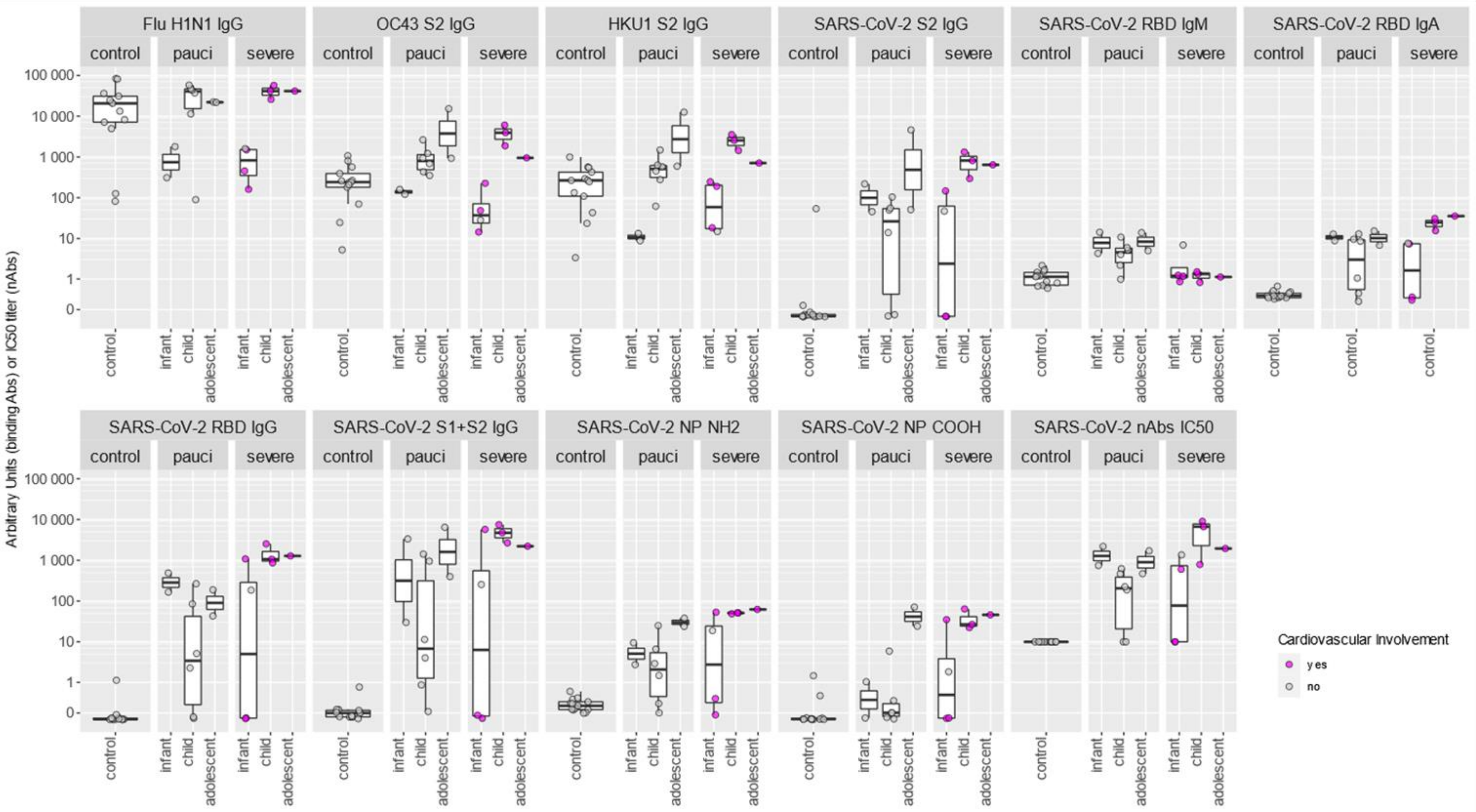

Supplementary Figure S1. Antibody responses in paediatric COVID-19 patients and controls.

In each panel the corresponding antibody levels are shown in patients stratified by disease severity and age. Circles correspond to each subject levels expressed as arbitrary units for binding antibodies or IC50 titer for neutralizing antibodies. Value distributions are summarized by boxplots showing median, interquartile range plus whiskers extending to ±1.96 fold the median. Presence of cardiovascular involvement is indicated by circle fill color.

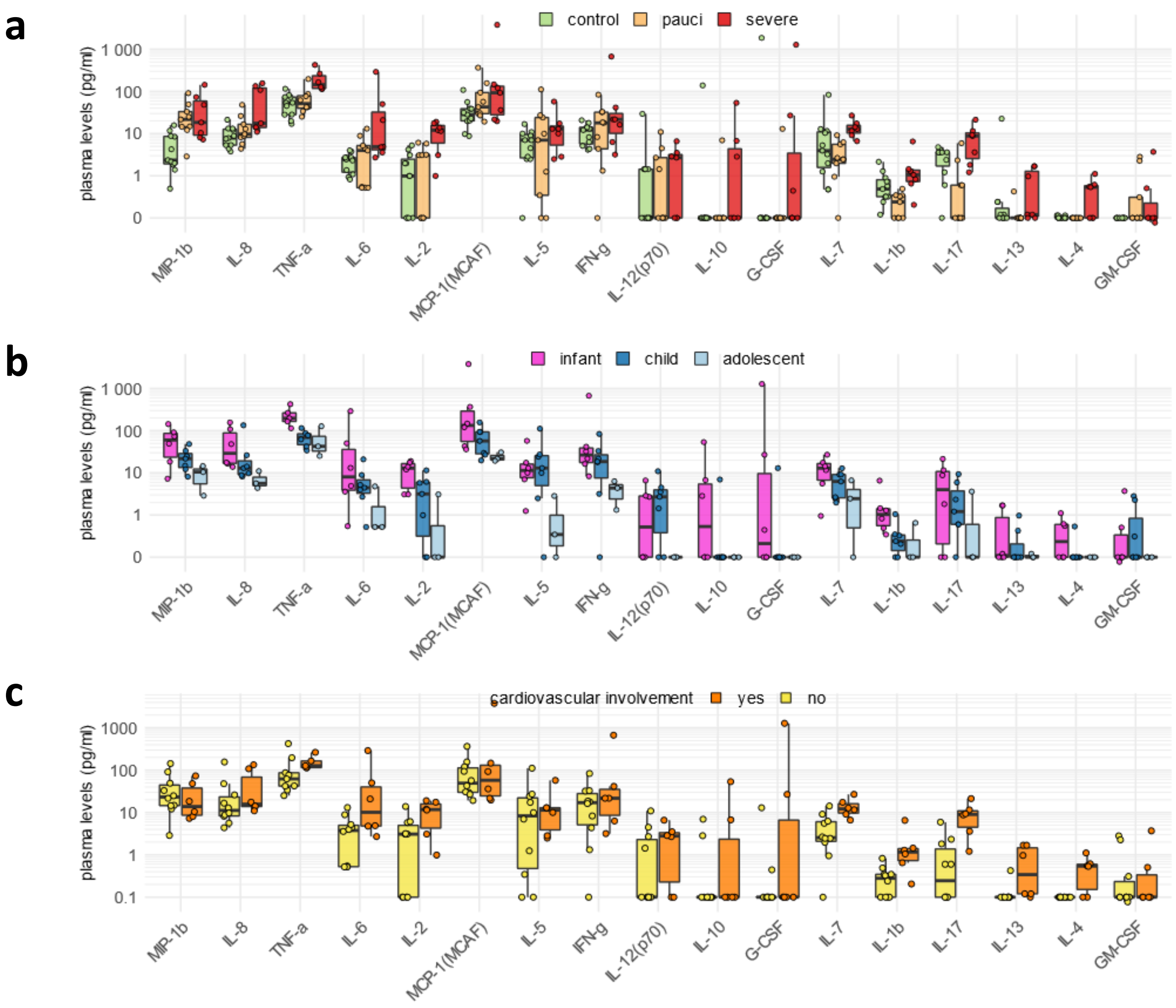

Supplementary Figure S2. Plasma levels of cytokines and chemokines in COVID-19 paediatric patients and controls.

The plasma concentration (pg/ml) of the corresponding cytokine/chemokine are shown in patients stratified by disease severity (Panel a), age (Panel b), and cardiovascular involvement (Panel c). Circles correspond to each subject levels. Value distributions are summarized by boxplots showing median, interquartile range plus whiskers extending to ±1.96 fold the median.

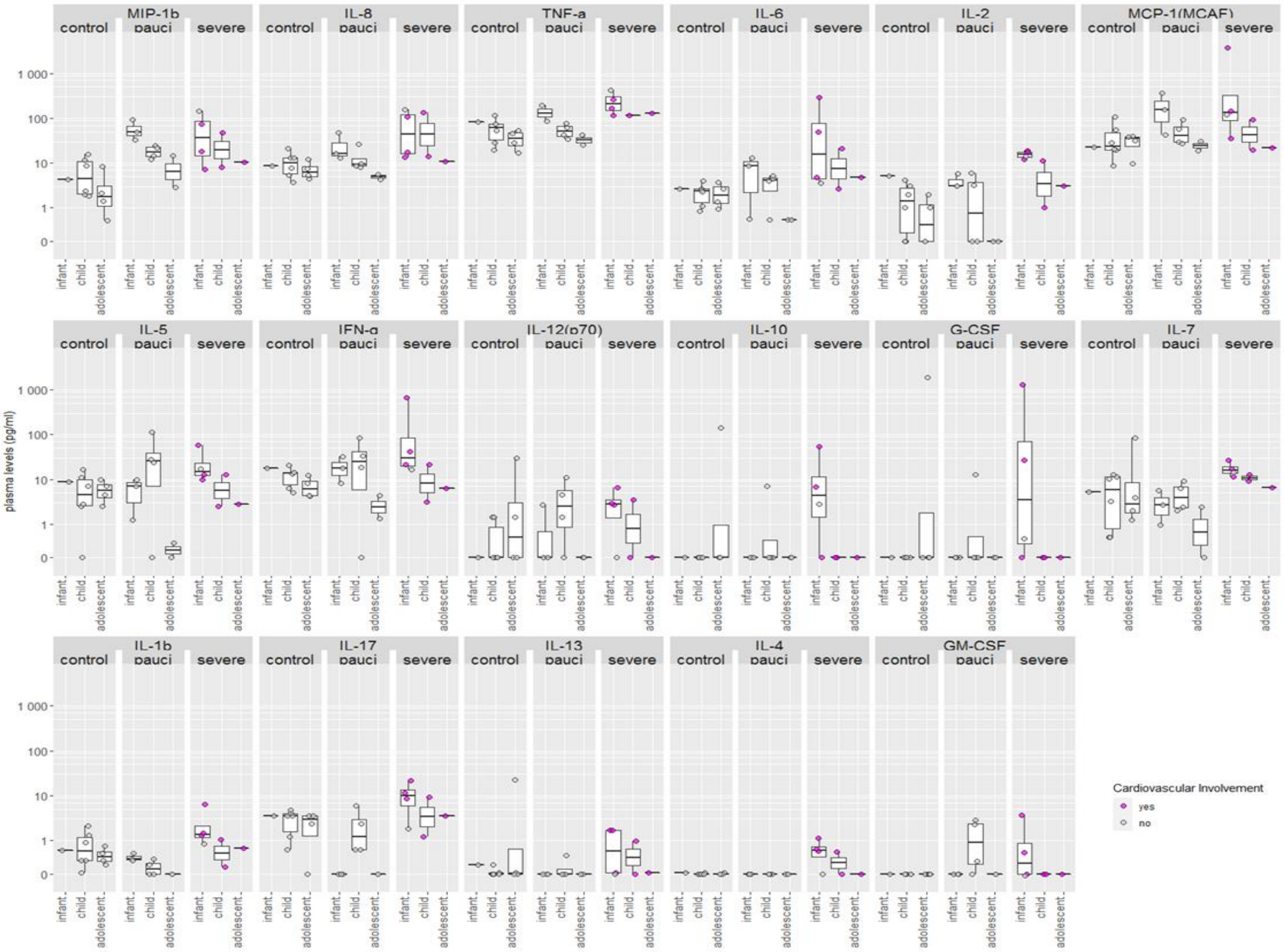

Supplementary Figure S3. Plasma levels of cytokines and chemokines in COVID-19 paediatric patients and controls.

In each panel the plasma concentration (pg/ml) of the corresponding cytokine/chemokine are shown in patients stratified by disease severity and age. Circles correspond to each subject levels. Value distributions are summarized by boxplots showing median, interquartile range plus whiskers extending to ±1.96 fold the median. Presence of cardiovascular involvement is indicated by circle fill color.

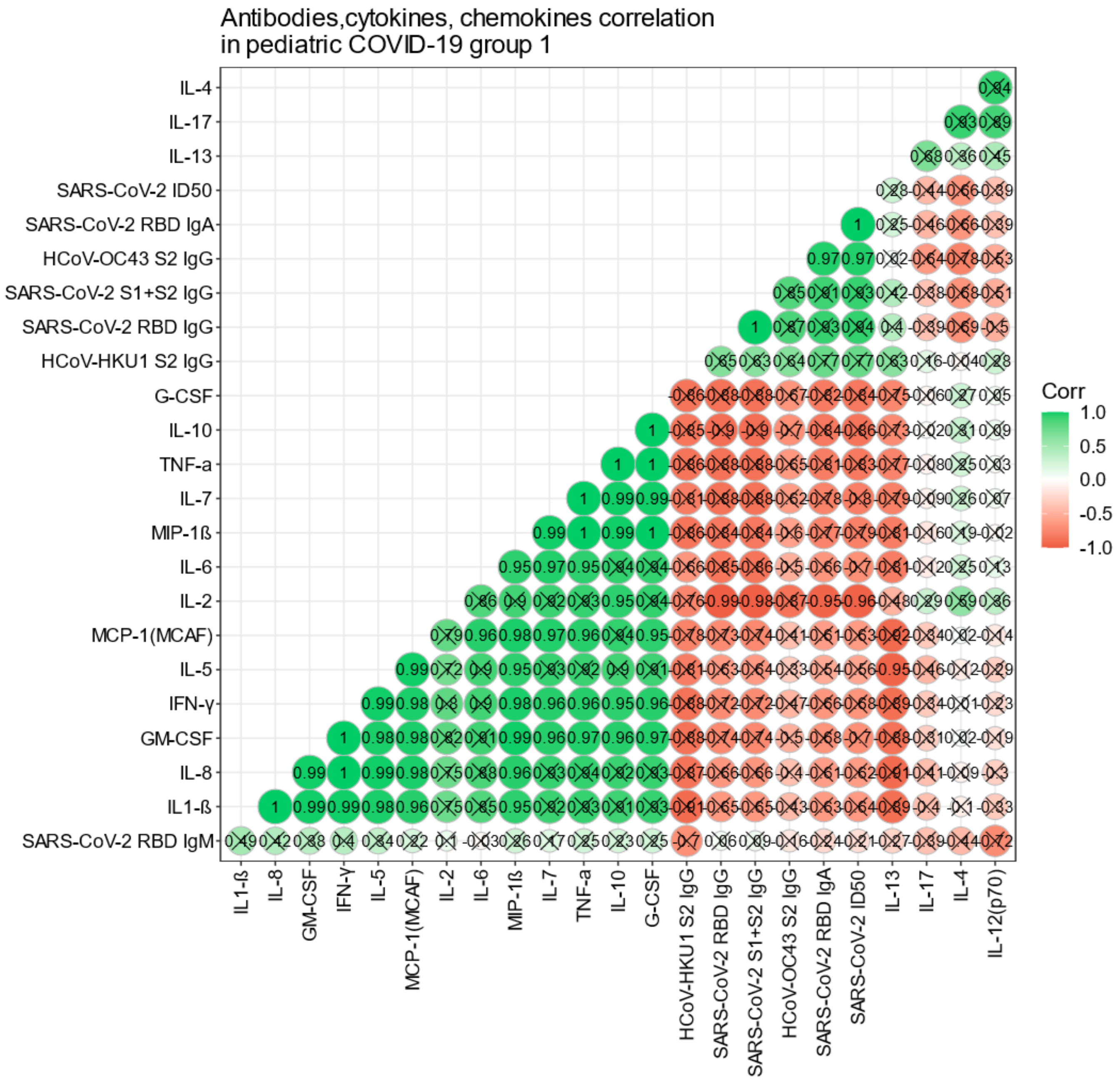

Supplementary Figure S4. Correlation of antibody responses, cytokines and chemokines in COVID-19 paediatric patients belonging to group 1.

Correloplot of quantitative antibody and cytokine/chemokine levels in patients from group 1. The circle fill color is proportional to the shown regression coefficients of the respective combination. Not significant correlations are marked by a cross.

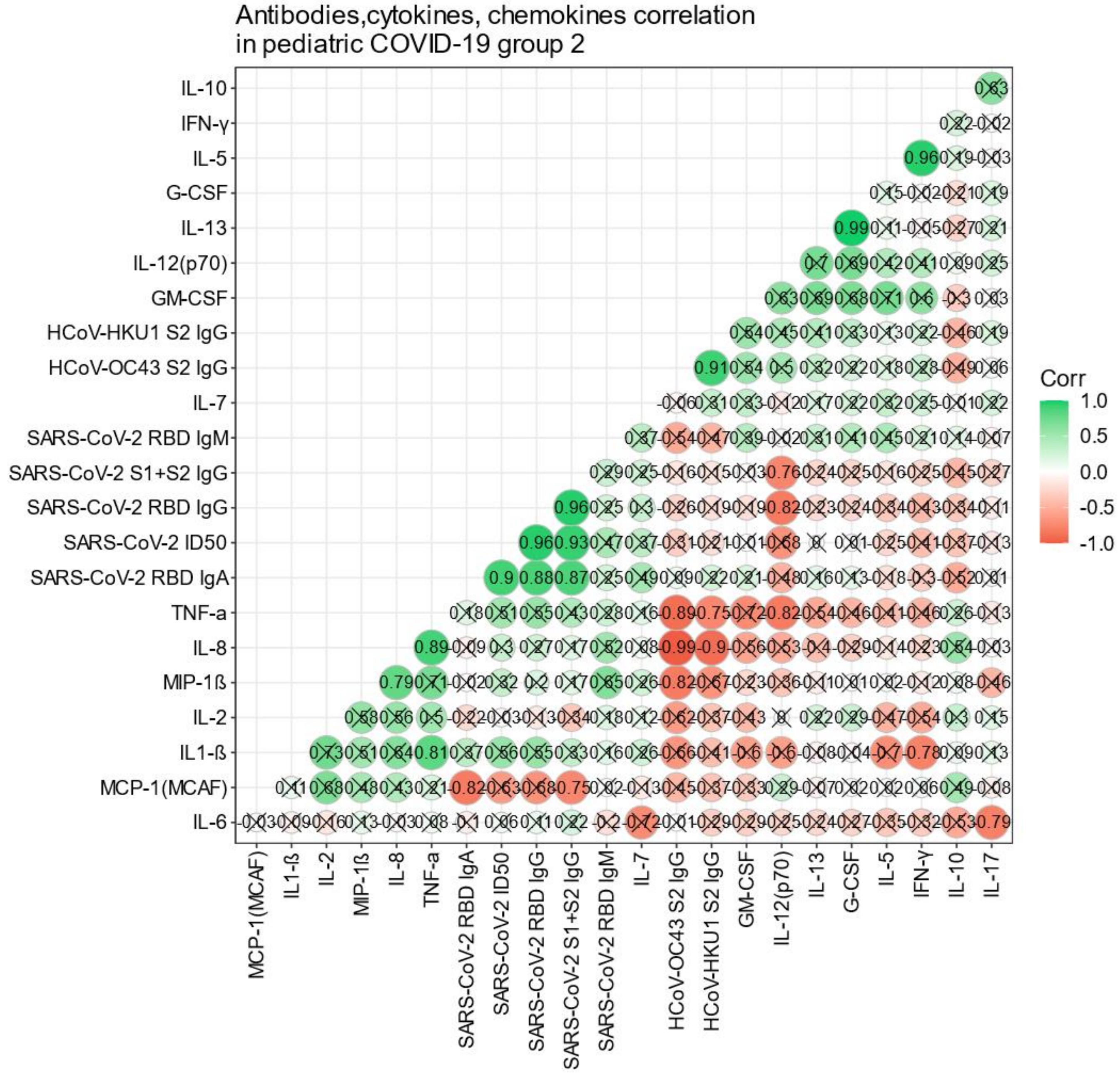

Supplementary Figure S5. Correlation of antibody responses, cytokines and chemokines in COVID-19 paediatric patients belonging to group 2.

Correloplot of quantitative antibody and cytokine/chemokine levels in patients from group 2. The circle fill color is proportional to the shown regression coefficients of the respective combination. Not significant correlations are marked by a cross.

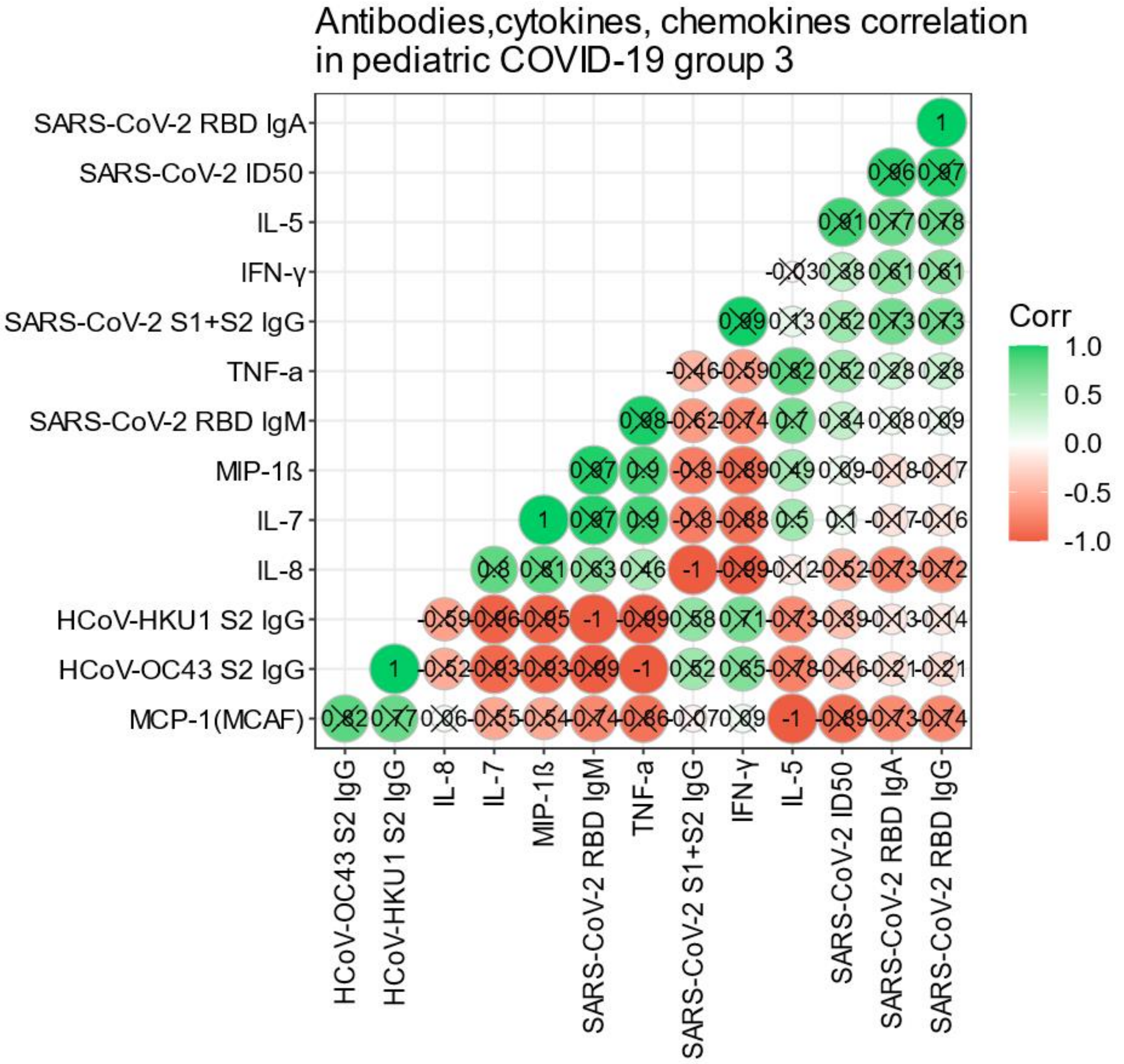

Supplementary Figure S6. Correlation of antibody responses, cytokines and chemokines in COVID-19 paediatric patients belonging to group 3.

Correloplot of quantitative antibody and cytokine/chemokine levels in patients from group 3. The circle fill color is proportional to the shown regression coefficients of the respective combination. Not significant correlations are marked by a cross.

a

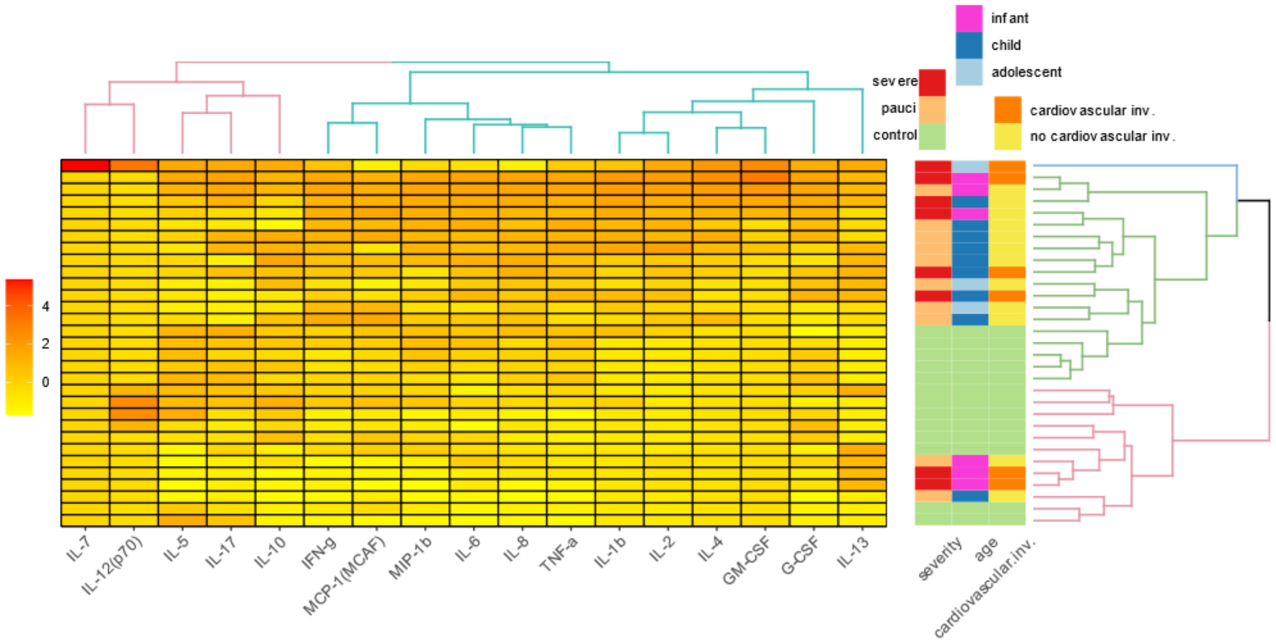

b

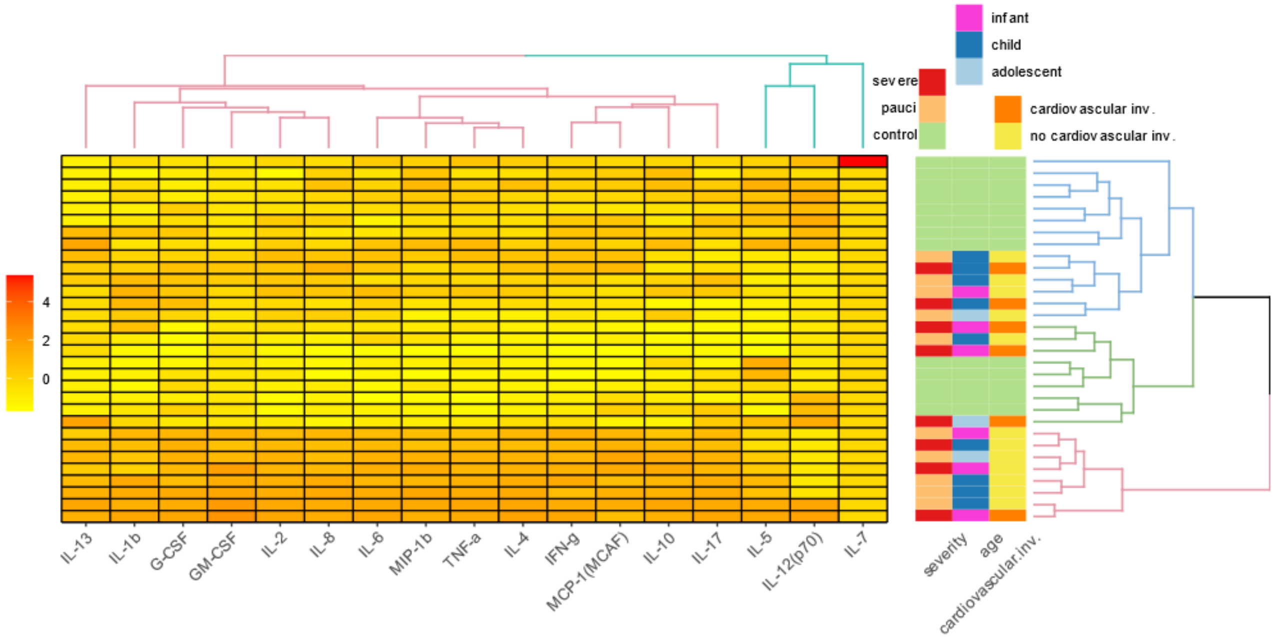

Supplementary Figure S7. Heatmap of cytokine/chemokine secretion by unstimulated or SARS-CoV-2-stimulated PBMCs in paediatric COVID-19 and controls.

Heatmap and clustering of patients and controls according to cytokine/chemokine secretion by unstimulated or SARS-CoV-2-stimulated PBMCs. Panel a: Heatmap of unstimulated secretion. Panel b: Heatmap of SARS-CoV-2-stimulated secretion.

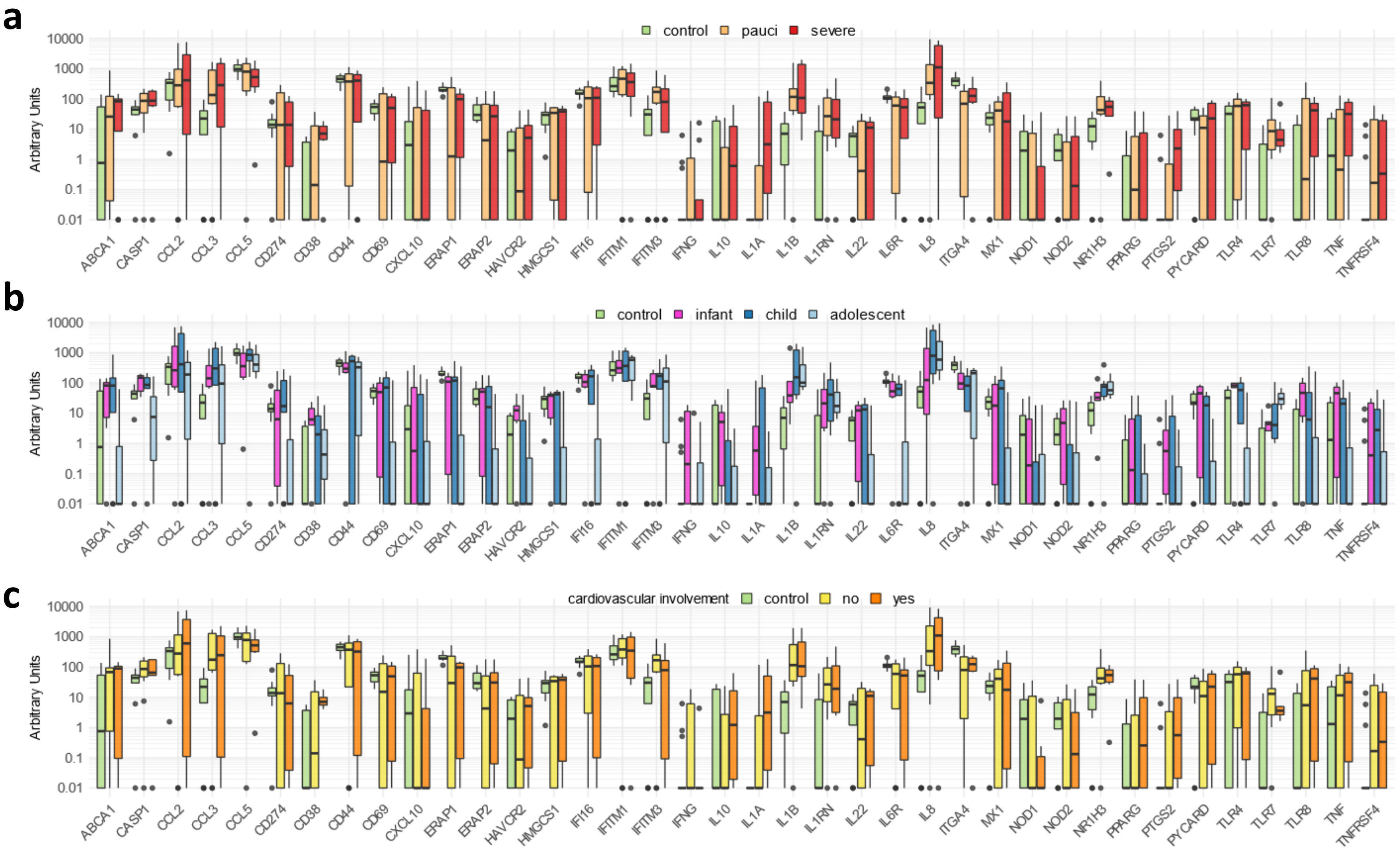

Supplementary Figure S8. PBMC cytokine/chemokine unstimulated gene expression in paediatric COVID-19 and controls

The mRNA levels of the corresponding genes in unstimulated PBMC are shown in patients stratified by disease severity (Panel a), age (Panel b), and cardiovascular involvement (Panel c). Value distributions are summarized by boxplots showing median, interquartile range plus whiskers extending to ±1.96 fold the median.

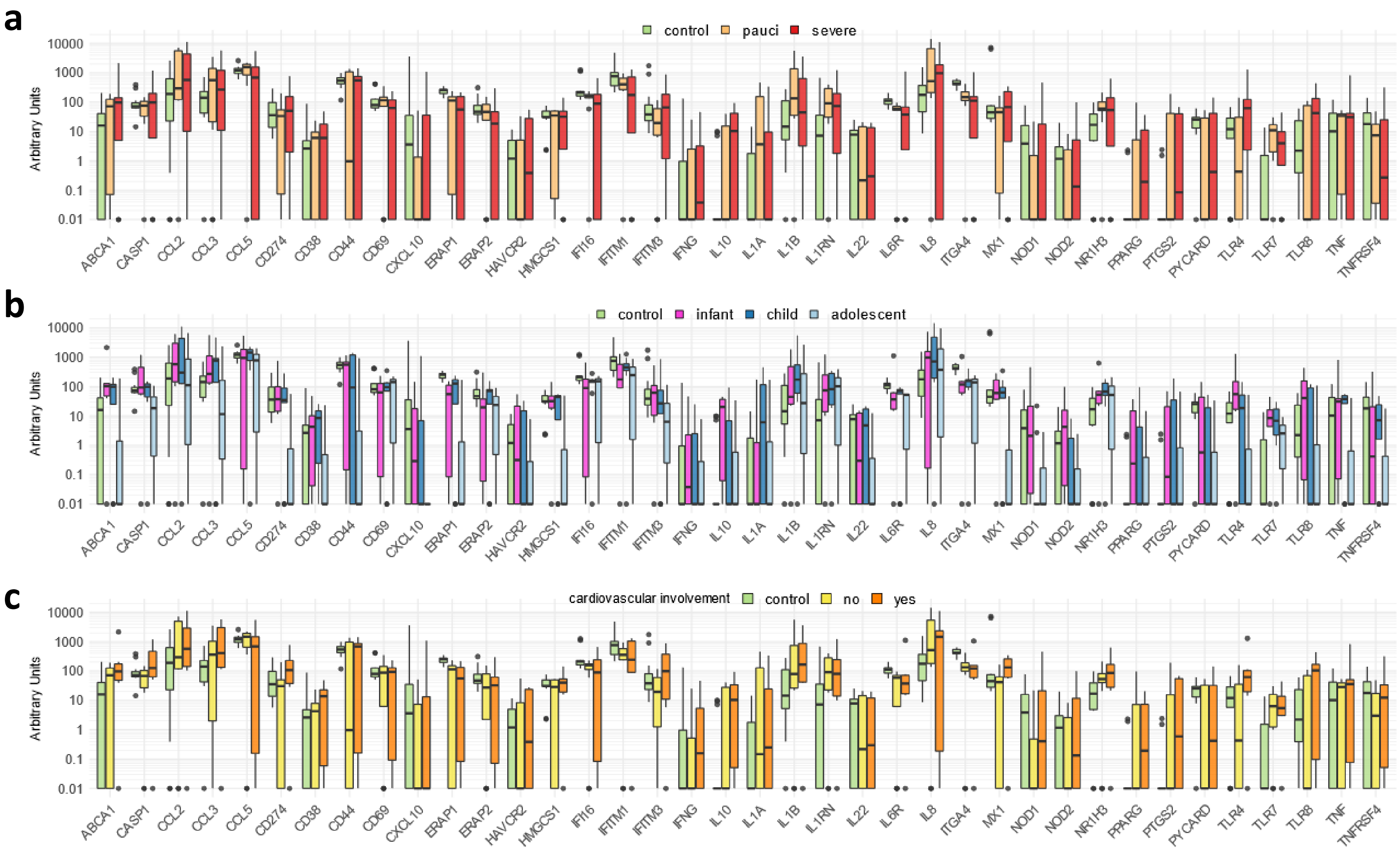

Supplementary Figure S9. PBMC cytokine/chemokine stimulated gene expression in paediatric COVID-19 and controls

The mRNA levels of the corresponding genes in SARS-CoV-2-stimulated PBMCs are shown in patients stratified by disease severity (Panel a), age (Panel b), and cardiovascular involvement (Panel c). Value distributions are summarized by boxplots showing median, interquartile range plus whiskers extending to ±1.96 fold the median.
